# Quality of outcome (QoO) in oral cancer patients: prospective perioperative analysis of patients’ resilience and satisfaction during inpatient stay at a University Medical Centre in Germany

**DOI:** 10.1515/iss-2024-0026

**Published:** 2024-08-02

**Authors:** Juliane Kröplin, Jil-Charlot Reppenhagen, Anke Hirsemann, Jan Liese, Bernhard Frerich

**Affiliations:** Department of Oral and Maxillofacial Surgery, University Medical Centre, Rostock, Germany; Department of Oncology, University Medical Centre, Rostock, Germany

**Keywords:** resilience, oncological outcome, perioperative management, tracheostomy management, patients’ satisfaction, prehabilitation

## Abstract

**Objectives:**

Quality assurance strategies for head and neck surgery can improve patient outcomes. This study aims a perioperative analysis of indicators in the quality of outcome in oral cancer patients with special focus on patient`s resilience and satisfaction before and after surgery.

**Methods:**

Patients with oral cancer (OCP) and indication for surgical resection were included in a prospective study. General and disease-related data as well as parameters of patient-specific resilience (RS-11 questionnaire) and physical strength (ECOG-Score) were collected. Importance and satisfaction using the nine items family/friends, sports activities/physiotherapy, participation in culture, professional perspectives, sexuality, enjoyable food, external appearance, social recognition and independence were analysed. The data collection took place pre- and postoperatively (mean: 10th postop day) during the inpatient stay.

**Results:**

Twenty-eight patients with oral cancer (OCP) were analysed (male: n=23, female n=5). The rate of R0 resections was 92.6 %. The average length of stay was 21 days. n=16 of all patients was tracheotomised postop (preop: n=0). n=0 patients confirmed psycho(onco)logical support preop (postop: n=19). Sports activities/physiotherapy was provided to n=3 patients preop (postop: n=21). The mean pre and postop pain perception was 3/10. The RS-11 score decreased significantly postoperatively (p=0.01). A significant loss of satisfaction was seen in the areas of enjoyable food (p<0.001), social recognition (p=0.01), external appearance (p=0.01) and independence (p<0.001).

**Conclusions:**

Supportive therapy measures to promote mental and physical fitness of oral cancer patients are part of inpatient care at our clinic. However, there is no standardised monitoring of these therapies. Digitally supported and standardised programmes to increase mental and physical fitness in both prehospital and inpatient care might lead to an improvement in the quality of outcome despite shortage of resources in terms of time, costs and personnel. A minimally invasive approach to tracheal cannula management and lymph node management in the treatment of OCP can help to reduce the length of stay in the intensive care unit.

## Introduction

With the introduction of the hospital transparency act, as part of the reform of hospital structures in Germany, the improvement and transparency of quality of outcome of patients with oncological diseases is gaining central importance [1].

Tumours in the head and neck area are among the most common cancers worldwide with an increasing incidence [2]. The vast majority of all malignant diseases of the oral cavity are squamous cell carcinomas. Risk factors are in particular the consumption of tobacco and/or alcohol as well as an infection with oncogenic viruses [3]. Men are affected more than twice as often as women and have a lower five-year survival rate of 52 % (women: 64 %). This can be explained in particular by the increased risk behaviour towards alcohol and tobacco [4].

The challenges in the treatment of head and neck cancer lie both in the anatomical characteristics of the head and neck region and in the complexity of interdisciplinary treatment consisting of surgery, radiotherapy and systemic therapy [3].

Quality assurance strategies are becoming increasingly important in order to optimise the quality of outcome [5], [6], [7]. It is essential to recognise that, in addition to the surgical outcome, patient-specific factors also have a significant influence on the recovery process [8], 9]. A deep understanding of the management of patients with aesthetic and functional deficits following maxillofacial surgery in the context of head and neck cancer is of particular relevance [10]. The social and interpersonal effects of diagnosis and surgical treatment are complex and influence the coping skills of those affected. These challenges range from fatalistic attitudes to post-operative anxiety and adjustment disorders [10].

Long-term rehabilitation takes into account aspects of restoring aesthetics, speech and chewing function [8], 11]. In this context, each patient has individual requirements and coping strategies, which have an impact on both survival and the patient’s quality of life [4]. Individual mental and physical fitness including patient-specific resilience are of particular interest [12]. Resilience refers to the ability to maintain or quickly recover to a healthy mental state during or after exposure to stressful life circumstances [13].

This study aims to analyse the quality of outcome in the treatment of oral cancer patients at a university medical centre for oral and maxillofacial surgery in Germany. Special consideration is given to patient`s resilience and satisfaction during the inpatient stay. For a structured evaluation of the quality of outcome, we defined the subareas *operating room (or) performance*, *perioperative management* and *patient’s perception*. In this way, we aim to further optimise quality assurance strategies in head and neck tumour surgery, creating transparency and thus contribute to a holistic improvement in patient care.

### Materials and methods

#### Patient collective and patient selection

The patient cohort was selected as part of a preoperative consultation on the day of admission to hospital before the operation. All patients with a histologically confirmed malignant tumour of the oral cavity and an indication for surgical resection were included. Exclusion criteria were non-consent and the inability of patients to consent. Furthermore, an existing psychiatric diagnosis led to exclusion from the study. Patients with a recurrence carcinoma were also excluded from the study. Data was collected pre- and postoperatively during the inpatient stay. The data collection period was 1.5 years (September 2022 to February 2024).

#### Demographics, patients and disease-related data

Age, gender and socioeconomic status were analysed. Socioeconomic status included school-leaving qualifications and monthly net income. Mental strength was analysed by patient-specific resilience, participation in psycho-oncological coaching and self-help groups.

Patient-specific resilience was determined using the RS-11 questionnaire [14]. The questions shown in Table 1 could be answered on a scale from 1 (strongly disagree) to 7 (strongly agree).

**Table 1:** RS-11 resilience-score.

If I have goals, I also pursue them
I usually manage everything somehow
It is important to me to stay interested in many things
I like myself
I can manage several things at the same time
I am determined
I retain an interest in many things
I often find something to be happy about
I can usually look at a situation from different perspectives
I can also overcome myself to do things that I don’t really want to do
I have enough energy to do everything I need to do

Physical strength was analysed by the Eastern Cooperative Oncology Group (ECOG) performance score (Table 2) [15], nicotin anamnesis and participation in sport activity.

**Table 2:** Eastern Cooperative Oncology Group (ECOG) performance score.

Score	Patient status
0	Fully active, able to carry on all pre-disease performance without restriction
1	Restricted in physically strenuous activity but ambulatory and able to carry out work of a light or sedentary nature, e. g. light house work, office work
2	Ambulatory and capable of all self-care but unable to carry out any work activities; up and about more than 50 % or waking hours
3	Capable of only limited self-care, confined to bed or char more than 50 % of walking hours
4	Completely disabled; cannot carry on any self-care; totally confined to bed or chair

The disease-related factors collected were the pathologically verified tumour nodulus and metastasis (TNM) stage, tumour stadium (I, II, III or IV) and the tumour location. In addition, the therapy was analysed taking into account the chosen type of reconstruction.

##### Supportive therapy measures

The use of supportive therapy measures including participation in self-help groups, psycho-oncological therapy, speech therapy and participation in sports programmes/physiotherapy was assessed perioperatively during the inpatient stay.

#### Indicators in the quality of outcome

Defined indicators of quality of outcome were analysed in the area of *operating room performance*, *perioperative management* and *patients*’ *perception*.

##### Operating room performance

To measure the surgical performance the R0-resection rate was assessed as well as postoperative complications (revision rate and mortality during the operation and the inpatient stay). At least one follow-up operation requiring anaesthesia during the inpatient stay, which served to remove a haematoma, a post-resection, an anastomosis revision, an emergency tracheotomy and a wound debridement, was counted as a revision.

##### Perioperative management

QoO indicators concerning perioperative management were the presence of a tracheostoma and a percutaneous endoscopic gastrostomy (PEG), the determination of the pain level, the performed lymphnode-management (SLNB, bilateral ND/unilateral ND), the length of intensive care (ICU) stay and overall inpatient stay. Patients with small tumours (T1 or T2) and the clinical and radiological exclusion of lymph node metastasis (N0) received a SLNB.

##### Patients’ perception

Importance and satisfaction of the nine items including *family/friends*, *sports activities/physiotherapy*, *participation in culture*, *career prospects*, *sexuality*, *enjoyment of food*, *physical appearance*, *social recognition* and *independence* was assessed using a Likert scale questionnaire (1=very important/satisfied to 5=not at all important/satisfied). The items were developed by the study directors on the basis of the four basic psychological needs according to Klaus Grawe: attachment, autonomy, self-esteem enhancement and pleasure gain [16].

#### Statistics

The data was analysed using IBM SPSS v27 (Armonk, NY, USA) software. Demographics general and disease-related data as well as data concerning surgical performance, perioperative management and patients’ perception were analysed descriptively. Statistical analysis for pre-post comparison was performed by a paired, two-tailed Student’s *t*-test. A value of p<0.05 was considered statistically significant.

### Results

#### Demographics, general and disease-related data

A total of 28 patients were included in the study. Table 3 shows all demographic, general and disease-related data which were queried preoperatively. The average age at diagnosis was seven years younger for men (65 years) than for women (72 years).

**Table 3:** Analysis of demographics, general and disease-related data.

Date	Subtype	n=28
Age	Mean	66
	Female – mean	72
	Male – mean	65
Gender	Female	5
	Male	23
Highest school qualification	No qualification	4
	Secondary school	9
	Intermediate school	8
	A-levels	3
	University	1
Relationship status	Partnership	16
	Single	12
Children	Yes	25
	No	3
Monthly net income (in Euro)	<999	5
	1,000–2,999	19
	3,000–4,999	1
	>5,000	0
	Unknown	3
Nicotin (including former nicotin abuse)	Yes	22
	No	5
	Unknown	1
	PY: ⌀ (n=12)	29
ECOG	Mean	1 (min 0 – max 3)

**Disease related data**		

Tumour stage (UICC-classification)	I	4
	II	8
	III	8
	IV	8
TNM stage	T1	4
	T2	8
	T3	8
	T4	8
	N0	20
	N1	3
	N2	4
	N3	1
	M0	28
	M1	0

##### Tumour entity and location

The most common tumour locations were the anterior and lateral floor of the mouth (n=11) and the tongue (n=6). With the exception of one adenocarcinoma of the gl. sublingualis, all tumours were classified as squamous cell carcinomas. n=2 carcinomas were located in the oropharynx. Further localisations were the mandibular mucosa (n=3), the buccal mucosa (n=2), the maxillary mucosa (n=1) and the maxillary sinus (n=1). Two tumours extended over several parts of the oral cavity.

##### Patient-specific resilience

**Figure 1:**
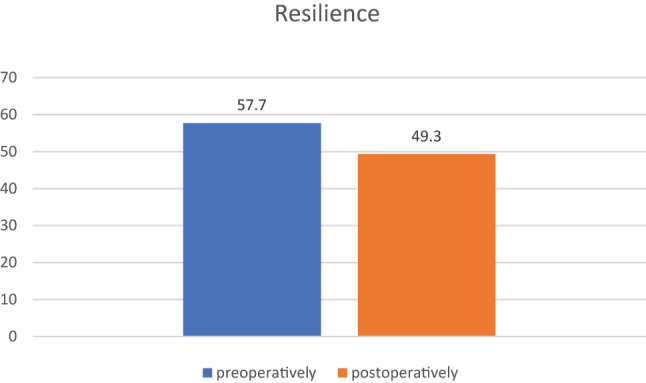
Patients’ resilience pre- and postoperatively.

Figure 1 shows the perioperative development of patient-specific resilience during the inpatient stay. The RS-score decreased significantly postoperatively (mean: preop 57.7/77, postop: 49.3/77; p=0.01).

##### Supportive therapy measures

n=0 patients confirmed psycho(onco)logical support preop (postop: n=21). Sports activities/physiotherapy was provided to n=3 patients preop (postop: n=19) (Table 4).

**Table 4:** Supportive measures (in %) pre- and postoperatively.

Therapy participation	Preoperatively, %	Postoperatively, %
Self-helping groups	0	3.6
Psychooncological coaching	0	67.9
Physiotherapy, sports activities	10.7	75.0
Speech/swallowing therapy	0	60.7

With regard to resilience, there was no significant difference between patients with and without supportive therapy measures during their inpatient stay. The perceived supportive measures also had no significant influence on the duration of the inpatient stay.

#### Indicators in the quality of outcome

##### Operating performance

The rate of R0 resections was 92.9 % (Rx: n=1, R1: n=1). The mortality rate during the operation was 0 %, but one patient died during the inpatient stay due to cardiovascular complications. A total of n=5 patients underwent revision surgery. A revision operation did not significantly prolong either the ICU stay (p=0.25) or the entire inpatient stay (p=0.40). Reconstruction using a microsurgically anastomised free flap was required in 23 cases. The most frequently used free flap was the radialis forearm flap (n=20). Other free flaps used were the fibula flap (n=2) and latissimus dorsi flap (n=1).

##### Perioperative management

The average length of stay was 21 days. n=16 of all patients was tracheotomised postop (preop: n=0). The mean postop pain perception was 3/10 (preoperatively 3/10). The number of patients with percutaneous endoscopic gastrostomy (PEG) was n=9. All patients received either a neck dissection or a sentinal lymph node biopsy. Of the neck dissections performed, 14 were bilateral and nine were unilateral.

The length of the ICU and inpatient stay is shown in Table 5. One patient from the SLNB group had an extreme value of 51 days of hospitalisation, so this value was not taken into account in the length of inpatient stay in Table 5. Taking into account the diagnosis-related-group (DRG) – relevant average maximum length of stay, this was exceeded by an average of two days (20.6 vs. 18.6). There is a significant reduction in ICU stays for patients without a postoperative tracheostoma and with sentinel lymph node biopsy (SLNB) instead of neck dissection (ND).

**Table 5:** Influence of tracheostomy and lymph node management on ICU stay (days) and length of inpatient stay (days).

Tracheostoma, n	ICU stay, days	Length of inpatient stay, days	Average length of stay (DRG)
Yes: n=16	3.1	22.5	19.4
No: n=12	1.6	17.8	17.6
	**p=0.04**	p=0.14	p=0.17

**Lymph node management**			

ND: n=23	2.8	21.8	19.1
SLNB: n=5	0.8	13.8	16.2
	**p=0.04**	p=0.06	p=0.08

P-values that show a significant difference in tracheostomy and lymph node management are marked in bold.

The distribution of tumour stage and tumour size is identical across the categories (yes/no) of a postoperative tracheostoma and the categories ND und SLNB. On average, however, SLNB was performed on smaller tumours.

##### Patients’ perception

With regard to the perioperative development of areas of life associated with psychological basic needs, there was no significant difference in the patients’ assessment of their importance. However, as shown in Table 6 a significant loss of satisfaction was seen in the areas of enjoyable food, social recognition, external appearance and independence.

**Table 6:** Importance and satisfaction concerning patients’ perception.

Item	Importance (p)	Importance (post)	Importance (p-value)	Satisfaction (pre)	Satisfaction (post)	Satisfaction (p-value)
Family/friends	1.3	1.4	0.42	1.4	1.8	0.13
Sports activities	3.0	3.3	0.09	2.5	2.8	0.13
Enjoyable food	1.6	1.3	0.06	**2.2**	**3.8**	**<0.001**
Culture	3.4	3.4	0.84	2.6	2.8	0.43
Professional perspectives	3.6	3.2	0.18	2.3	2.4	0.48
Social recognition	3.0	3.0	0.85	**2.2**	**2.7**	**0.01**
Sexuality	2.7	2.9	0.26	2.6	3.0	0.13
External appearance	2.1	2.5	0.07	**2.4**	**3.2**	**0.01**
Independence	1.5	1.4	0.80	**1.8**	**3.3**	**<0.001**

Values that show a significant difference in the pre- and postoperative comparison are marked in bold.

### Discussion

#### Demographics, general and disease-related data

Both the gender distribution and the average age are representative of OCP [15]. The same applies to the socioeconomic status, which is considered to be rather low overall [17]. This is reflected both in the demographic data of our patient population and in our patients’ perceived low importance of participation in culture. The fact that low socioeconomic status is associated with lower health literacy has been described [18]. Tumour diseases also occur earlier and more frequently in patients with a low socioeconomic status. In addition, they benefit less from therapeutic measures and have a lower overall survival rate [17], 18]. As a result, target group-orientated educational approaches to strengthen health literacy could help to reduce the incidence of oral cancer [17]. It is important to identify suitable communication channels at a structural level and to facilitate regular access to medical check-ups. Screening procedures that are easy to use and low-threshold in terms of access could be a suitable method. Currently, the majority of oral cavity cancers are diagnosed by dentists in private practice as part of routine examinations [2]. The further development of digital applications also has the potential to contribute to enabling independence from time and place. Digital literacy must be considered consecutively under the aspect of health literacy.

The patient clientele we examined is on average limited in physically strenuous activity but able to walk and perform light or sedentary work (ECOG 1). This means that adapted sports programmes are generally possible in the perioperative setting.

Only three of our patients had already done sports before the operation. This is remarkable from the point of view that such measures can already be useful in a pre-operative setting to improve the quality of outcome [19], 20]. For concrete implementation in the perioperative setting, however, an individual patient assessment should be carried out, including the collection of objective parameters of physical endurance – such as the determination of VO2max in the endurance performance range.

None of the patients we analysed had taken advantage of pre-operative mental coaching. The resilience score recorded preoperatively almost corresponds to the results of the validation study by Schumacher et al. [14]. This is an indication that the transmission of the diagnosis itself at the time of hospitalisation has no negative influence on the patient’s divergent resilience. The perioperative analysis, on the other hand, shows a significant decrease in resilience. Consequently, it makes sense to look into the possibilities of resilience interventions.

As current literature shows, the effect of resilience interventions on acute cancer patients is particularly high [13]. Patients can benefit most in the long term in the phase immediately after diagnosis and in the context of somatic therapies [13]. This aspect should be taken into account in any planned intervention.

#### Indicators in the quality of outcome

The surgeon’s experience and the centralisation of healthcare services have a decisive influence on the quality of outcome of surgical procedures [21]. Adequate further training of (future) tumour surgeons is therefore of particular importance [22]. In addition to practical skills, interprofessional skills and the ability to work in a team also play an important role in the treatment of tumours in the head and neck area. For example, the microsurgically anastomosed free flaps most frequently used at our clinic for reconstruction after tumour surgery (radial forearm free flap) allow the possibility of a two-team approach. This is a decisive advantage in terms of process quality against the background of reduced operating room time.

Airway management after tumour surgery in the head and neck region is an essential part of perioperative management [23], 24]. The aim of an ideal airway is to avoid long-term morbidity, to support the recovery of physiological function of speech and swallowing and to enable early discharge from hospital [25]. In terms of quality management, a deliberate indication for tracheostomy or its avoidance is definitely expedient. Firstly, this reduces the duration of the operation. It must also be taken into account that the tracheostomy represents an independent risk factor for postoperative complications such as bleeding, obstruction and dislocation of the cannula, airway obstruction, fistula formation and pneumonia [24].

In the patient population we examined, none of the patients had a tracheostomy at the start of surgical treatment. However, advanced tumour diseases with indication for microsurgical reconstruction in the oral cavity pose risks for a safe airway due to airway and flap oedema, haematoma formation and the risk factors typical for the patient clientele, such as chronic nicotine abuse [26]. Consecutively, the indication for an elective tracheostomy arises. More recent approaches recommend one-night intubation in the sense of delayed extubation. The procedure depends on the surgeon’s surgical experience and the possibilities of postoperative monitoring [25].

In our results, a significantly reduced ICU stay was observed in non-tracheostomised patients. Our results correspond to the results of current literature dealing with the possibilities of efficient perioperative management [27]. Coyle et al. demonstrated the safety of replacing tracheostomies with overnight admission to the ICU, which resulted in fewer respiratory complications and shorter ICU stays (3.7 vs. 1.4 days) [27].

A retrospective study from 2017 also showed that the majority of affected patients (60 %) would have been “very happy” to do without a tracheostomy. The main reasons lie in the areas of fear and communication [28]. The presence of a tracheostoma reduces the quality of life [29], this means that the responsible clinics have to deal with strategies for avoiding tracheotomies and the possibilities of early decannulation in tracheotomized patients in order to achieve optimum quality of outcome.

With regard to lymph node management, the minimally invasive SLNB approach is established at our clinic. Patients with small tumours (T1 and T2) and the clinical and radiological exclusion of lymph node metastasis (N0) receive this treatment regime. The aim is to avoid a significantly more invasive neck dissection. Taking into account the patient clientele analysed by us an SLNB was performed in five cases. Neck dissection was prevented in four cases. The results show the particular importance of this diagnostic procedure. Additionally, patients with SLNB were in the intensive care unit for a significantly shorter time.

The results of our tracheostomy and lymph node management emphasises the importance of a minimally invasive approach to surgical treatment.

In addition to the assessment of objective criteria, the assessment of the quality of outcome should ideally also include the perspective of the patients concerned [19].

Our results show that surgical therapy influences aspects of our patients’ basic psychological needs. This specifically concerns satisfaction and not the importance of defined areas of life.

The dissatisfaction in the area of enjoyable food is hardly surprising. Swallowing and speech disorders are typical symptoms of patients with head and neck tumours [19]. These can be caused by the disease itself, by an operation or by adjuvant therapy measures such as radio(chemo)therapy. Here, it is important to further adapt the speech therapy measures to the patient’s needs and to support the rapid recovery of possible speech and swallowing functions through adequate tracheal cannula management.

The extent to which this should be addressed in a pre-therapeutic setting is the subject of current debate. In a literature review by Loewen et al. on the prehabilitation of patients with head and neck tumours from 2021, the most commonly practised swallowing exercises such as the Mendelsohn manoeuvre and effortfull swallowing were described [19]. However, no evidence-based regime could be identified for the frequency of implementation. It might be expedient to base the choice of intensity of speech therapy on the outcome (strength, endurance, both), analogous to rehabilitation concepts [30].

The other areas of patient reported dissatisfaction could, for example, be countered by preoperative therapy approaches based on a holistic prehabilitative treatment concept to strengthen the mental and physical fitness of patients. Even if our results represent a normal psychological reaction to an exceptionally stressful event, existing potential – particularly in the area of behavioural therapy – should be further exploited.

#### Digital approaches for optimizing

The existing and worsening shortage of healthcare professionals must be taken into account in the implementation of supporting programmes. The deployment of qualified personnel is also time-consuming and cost-intensive. From this point of view, digital solutions offer an interesting therapeutic approach for therapeutic measures to increase mental and physical strength in the context of prehabilitation, inpatient treatment and rehabilitation in surgery [31], 32].

As our results show, some patients have different prerequisites for the use of supportive measures. Both the wide spread in the ECOG score and the diversity in terms of age and stage of disease indicate the need for preoperative risk stratification.

When applying digital solutions, it must still be taken into account that patients with a low socio-economic status and advanced age in particular require special motivation and support to achieve adequate adherence [31].

#### Limitation

The present study is a single-centre study design. Limitations result from the small number of patients. In determining the quality of outcome, we present data during the inpatient stay. The postoperative analysis is limited exclusively to the average 10th postoperative day. With regard to patient-specific resilience, we cannot make any statement about how this was before the diagnosis was made. This study does not claim to be a general statement on the quality of outcomes in head and neck tumour surgery. Further studies in a multicentre study design are necessary for a more detailed analysis. Of particular interest is the further postoperative course in the outpatient setting.

### Conclusions

Supportive therapy measures to promote the physical and mental fitness of oral cancer patients are part of inpatient care at our clinic. However, there is no standardised monitoring of these therapies. Structured but patient individualised programmes to increase mental and physical fitness in both prehospital and inpatient care could lead to an improvement in the quality of outcome – such as higher resilience, a greater patients’ satisfaction in terms of their basic psychological needs and a shorter hospitalisation. This might also improve the comparability of treatment strategies and postoperative courses. Digital solutions are innovative approaches to tackling the shortage of resources in terms of time, costs and personnel. A minimally invasive approach to tracheostomy management and lymph node management in the treatment of OCP can help to reduce the length of stay in the intensive care unit.
